# Supplementary material for: Genetic Profiles of Aggressive Variants of Papillary Thyroid Carcinomas
Source: Cancers (Basel). 2021 Feb 20;13(4):892. doi: 10.3390/cancers13040892 (PMC7924361; doi:10.3390/cancers13040892)
Supplement: Supplementary file 1 [file cancers-13-00892-s001.pdf]

cancers

Genetic Profiles of Aggressive Variants of Papillary Thyroid Carcinomas

Meihua Jin, Dong Eun Song, Jonghwa Ahn, Eyun Song, Yu-Mi Lee, Tae-Yon Sung, Tae Yong Kim, Won Bae Kim, Young Kee Shong, Min Ji Jeon and Won Gu Kim

Table S1. List of 50 target genes.

TERT promoter	*BRAF*	*NRAS*	*HRAS*	*KRAS*
E1F1AX	*TP53*	*RB1*	*NF1*	*NF2*
PTEN	*PI3K3CA*	*ATM*	*MEN1*	*ARID1B*
TSHR	*MED12*	*RBM10*	*ARID2*	*POLE*
PPM1D	*STK11*	*CHEK2*	*ZFHX3*	*BDP1*
KMT2A	*KMT2C*	*TG*	*EZH1*	*APC*
AKT1	*SPOP*	*ALK*	*RET*	*PIK3C2G*
PIK3CG	*AKT3*	*TSC2*	*MTOR*	*ARID1A*
ARID1B	*ARID5B*	*SMARCB1*	*PBRM1*	*ARTX*
KMT2D	*MSH2*	*FGFR3*	*PDGFRB*	*ERBB3*

Table S2. Comparison of genetic profile of TCV-PTC and CCV-PTC.

Pathological Subtypes of Thyroid Cancer	TCV-PTC	CCV-PTC	*p*-value
**Driver genes**	92%	82%	0.78
BRAF	92%	73%	0.33
NRAS	0%	9%	0.69
***TERT* promoter**	17%	18%	0.99
**Tumor suppressor genes**	25%	18%	0.99
ZFHX3	17%	9%	0.94
TP53	4%	0%	0.99
CHEK2	4%	9%	0.99
**Key pathways and functional groups**	33%	36%	0.99
HMTs	8%	18%	0.78
SWI/SNF complex	17%	9%	0.94
PI3K/AKT/mTOR pathway	13%	9%	0.99

Abbreviation: TCV, tall cell variant; PTC, papillary thyroid carcinoma; CCV, columnal cell variant; HMTs, histone methyltransferase.

Table S3. *BRAF* and *NRAS* mutation in TCV-PTC patients.

TCV-PTC	TCV-PTC of PTC-TCGA (*n* = 29)	TCV-PTC of Current Study (*n* = 25)
BRAF	97%	92%
NRAS	0%	0%

Abbreviation: TCV: Tall cell variant, PTC: papillary thyroid carcinoma, TCGA: The Cancer Genome Atlas.
